# Effectiveness of New Maternal Mental Health Service ‘Thrive’ in the Treatment of PTSD Symptoms Arising From Birth Trauma and Perinatal Loss

**DOI:** 10.1192/bjo.2022.386

**Published:** 2022-06-20

**Authors:** Helen Crook, Athena Duffy, Bosky Nair, Rose Waters

**Affiliations:** Kent and Medway NHS and Social Care Partnership Trust, Maidstone, United Kingdom

## Abstract

**Aims:**

The NHS Long-Term Plan includes the perinatal mental health objective: by 2023/24 ‘Maternal Mental Health Services’ will be available across the country to provide psychological therapy for those who experience mental health difficulties directly arising from birth trauma and or/perinatal loss. We achieved early implementer status via application to NHS England and, using transformation funding received, ‘Thrive’ was piloted in East Kent. A gap in service provision was identified: some existing primary care services provide intervention for this cohort, however some people remain in psychological distress but do not meet the criteria for specialist perinatal mental health secondary care services; these secondary care services are not commissioned to support those who have experienced perinatal loss. Thrive is co-delivered by a mental health trust and acute healthcare trust; NICE recommended psychological interventions are provided by Psychological Therapists, Specialist Mental Health Midwives and a Peer Support Worker. The aim of this project was to evaluate the effectiveness of the Thrive pilot in reducing PTSD symptomology whilst also collating feedback from patients, their families and healthcare staff across the maternity system, in order to adapt the service offer for full county rollout.

**Methods:**

40 people who received care from Thrive from 11th January 2021 to 31st December 2021 were included in this evaluation.

Data were collected retrospectively at the end of each period of care via:

Clinical outcomes measures (quantitative):

- PCL-5: a 20-item self-report measure assessing the 20 DSM-5 symptoms of PTSD.
- CORE-34: a universal method of establishing well-being and risk.
- HoNOS (Health of the Nation Outcomes Scales): a measure of the health and social functioning of people with severe mental illness.

Patient Satisfaction Survey (qualitative).

**Results:**

- 100% of patients improved following Thrive intervention: PCL-5 (significant change = a reduction in score by 10–20 points has been met) / CORE-34 (clinically significant change =score above 10 initially and below 10 after intervention).
- Clinical improvement: HoNOS = 100% of patients improved following Thrive intervention.

**Conclusion:**

Evaluation has evidenced the effectiveness of Thrive in successfully treating those with PTSD symptomology arising from their maternity experience. Post-treatment measures indicate that the level of trauma symptomology and the impact of psychological distress on the functioning of patients who have received intervention from Thrive has reduced to a sub-clinical level in all cases.

